# Necrotizing Fasciitis in a Pediatric Patient Caused by Lancefield Group G Streptococcus: Case Report and Brief Review of the Literature

**DOI:** 10.1155/2011/671365

**Published:** 2011-12-27

**Authors:** John Rausch, Marc Foca

**Affiliations:** ^1^Division of General Pediatrics, Department of Pediatrics, Columbia University Medical Center, New York, NY 10032, USA; ^2^Division of Pediatric Infectious Diseases, Department of Pediatrics, Columbia University Medical Center, New York, NY 10032, USA

## Abstract

We report a case of necrotizing fasciitis with an accompanying toxic shock syndrome caused by Group G Streptococcus in a pediatric patient with a lymphatic malformation. Pediatricians need to be aware of the possibility of such infections, especially in those with vascular/lymphatic malformations, as early treatment is critical for survival.

## 1. Introduction

Necrotizing fasciitis (NF) is a potentially life-threatening soft tissue infection that is characterized by necrosis of the fascia and subcutaneous tissue and fat. The prognosis depends upon accurate diagnosis and early surgical debridement. This is often difficult due to the lack of initial physical signs. NF is most common in immunocompromised hosts but may also occur in healthy patients without apparent antecedent injury. It is usually caused by either Group A streptococci or a polymicrobial, synergistic infection. There are also emerging monomicrobial causes, including those caused by non-Group-A Streptococci such as groups B, C, and G. We report a case of necrotizing fasciitis with an accompanying toxic-shock syndrome caused by Group G Streptococcus in a pediatric patient with a lymphatic malformation. 

## 2. Case Report

The patient was a 2-year-old boy with a history of a prenatally diagnosed vascular malformation with both lymphatic and capillary components, involving the left retroperitoneum, abdomen, pelvis, and thighs. He had had previous debulking surgery soon after birth and another surgery in the first year of life to remove a lymphatic tumor that had developed over the left buttocks. Since then, he had received multiple rounds of sclerotherapy. There was a history of three previous admissions related to overlying cellulitis of the area and possible infection of the malformation given increased swelling and tenderness; although no organism had ever been cultured, including fluid aspirated from the mass. His first admission (1 year prior to this admission), was at an outside institution and the patient was treated with ceftriaxone and then oral cefixime. During his second admission (4 months later), the patient was initially treated with ampicillin/sulbactam, vancomycin and gentamycin for anaerobic, Gram-positive, and Gram-negative coverage. He was discharged home on levofloxacin for a total of 14 days. On his last admission prior to this presentation, the patient was treated with levofloxacin, completing a 14-day course. He presented to the emergency room (ER) on the day of the current admission with a 1-day history of fever (Tmax 103.5) and 1 episode of vomiting. The patient's mother reported that his malformation had increased in size and seemed more tender. The patient had gone for scheduled sclerotherapy, but it was cancelled due to the fever. In the ER, he was noted to be dehydrated and his lymphangioma was tender throughout, particularly in the perineum, but it was not erythematous. Complete blood count revealed a white blood cell count (WBC) of 1.5 with 47% neutrophils, 14% bands, 26% lymphocytes, and 11% monocytes. Platelets were 328 and hemoglobin and hematocrit were 11.9 and 36.1, respectively. The patient had previously had a normal white blood cell count. Erythrocyte sedimentation rate (ESR) was 10, but C-reactive protein was 265. He did not have hematuria, but urinalysis showed 1 + albumin, trace ketones, and 3 + blood. He was given an IV fluid bolus, started on levofloxacin, and admitted to the hospital. 

On hospital day 2 the patient was noted to be grunting in pain with increased tenderness over the perineum, despite a similar physical appearance of the malformation. On hospital day 3 the patient developed a facial rash and peripheral edema, as well as edema of the lower abdomen. His blood pressure dropped to 70's/40's and he received crystalloid and albumin. The infectious diseases service was consulted and antibiotics were changed to piperacillin/tazobactam and tobramycin empirically for pseudomonas and to cover for any organism with possible levofloxacin resistance, given his previous treatment. The patient's blood pressure dropped again to 60's/30's. Laboratory values at the time revealed: D-Dimer 3.32, prothrombin time 23, international normalized ratio 1.93, partial thromboplastin time 44.5, sodium 131, bicarbonate 8, WBC 5.2, hematocrit 26.2, and platelets 50. The patient was transferred to the pediatric intensive care unit (PICU) for presumptive septic shock with respiratory distress and progressive edema. He was intubated on admission to the PICU. 

On hospital day 5 the patient had worsening edema and erythema of the perineum. A CT scan was performed which did not show any definite abscess, but he was taken to the operating suite where bilateral fasciotomies and debridement of necrotic tissue in the perineum was performed. A diverting colostomy was established and drains were left in place. Chest X-ray showed a large right pleural effusion and a chest tube was placed. Metronidazole was added to improve anaerobic coverage.

On hospital day 7 the patient returned to the operating room for further debridement. At this time he had clindamycin added to his antibiotic regimen to combat a potential “inoculum effect” and in case there was toxin production. IVIG 2 g/kg was also given, because signs and symptoms were consistent with a superimposed toxic shock picture. Continuous veno-venous hemofiltration (CVVH) and high-frequency oscillating ventilation were initiated. Several days later pure cultures of *β* hemolytic Streptococcus Group G, *S. dysgalactiae* subspecies *S. equisimilis* was isolated from wound and from stool. The stool culture was collected on hospital day 1. The wound culture from the necrotic perineal tissue was collected on hospital day 7 after his second surgical procedure. There was no susceptibility testing done and no further typing of either sample.

The patient returned to the operating suite several times for debridement, Broviac placement, and ultimately for wound closure. Over the next 5 weeks he was weaned off CVVH, the ventilator, and restarted on regular feeds. He was ultimately treated with a six-week course of piperacillin/tazobactam. It was felt that such a long duration of therapy was necessary given the patient's critical condition and his multiple trips to the operating room. He was discharged after 6 weeks.

The patient's father signed a HIPAA consent form authorizing this case report. 

## 3. Discussion

### 3.1. Necrotizing Fasciitis

Necrotizing fasciitis (NF) was described in the 5th century BC by Hippocrates [1]. It has had numerous appellations, including hospital gangrene, phagedena gangrenosum, progressive bacterial synergistic gangrene, and Fournier's gangrene [2]. The currently used term, NF, was first championed by Ben Wilson in 1952 as the most descriptive of this infectious process [3]. The process is marked by necrosis of the superficial fascia, neutrophil infiltration of the deep dermis and fascia, thrombosis of the cutaneous microcirculation, and the presence of the infecting organism in the necrotic tissue [2, 4, 5]. It must be distinguished from cellulitis, which lacks necrosis, and an abscess, which is a localized purulence as compared to the diffuse necrosis seen in NF [5]. These infections most often occur on the extremities, abdomen, and in the perineum, but can occur anywhere on the body [5].

NF is a relatively rare condition, with an incidence of 500 to 1500 reported cases caused by Group A Streptococcus per year in the US [2, 6]. There are numerous risk factors, but the immunocompromised are particularly susceptible. Some of these conditions include diabetes mellitus, cancer (particularly chronic lymphocytic leukemia), HIV, peripheral vascular diseases, renal or liver impairment, chronic corticosteroid use, chronic skin ulcers, recent surgery, and obesity [5, 7]. NF usually starts at a site of trauma, often inapparent, which may be a minor puncture wound, blunt trauma, or a surgical scar [5].

 NF can be divided into two distinct groups based upon the causative organisms [2, 7, 8]. Type I is a polymicrobial infection caused by non-Group-A Streptococci (including Groups B, C, and G), aerobic organisms (including *Enterobacter*, *E. coli*, *Klebsiella*, and *Pseudomonas*), and anaerobic organisms (including Bacteroides and Clostridium). This type is most often found in patients who are immunocompromised. Type II infections are usually caused by *Streptococcus pyogenes* alone or with Staphylococci. These can occur in individuals with no underlying comorbidities [2, 7, 8]. There are other monomicrobial infections as well, including marine *Vibrio* supspecies (Gram-negative rods), usually associated with sea water or marine animal exposure. Chronic liver disease is a reported as a predisposing factor [9]. In addition, there have been increasing numbers of infections caused solely by non-Group A Streptococci [10–16].

In a study by Wong et al., the typical clinical presentation of patients with NF was elucidated. Their patients usually presented with exquisite pain, swelling, and fever. The only initial signs were often tenderness, erythema, and warm skin. Additionally, pain out of proportion to clinical findings was the most common initial symptom [2]. Other studies have shown a flu-like appearance during this initial stage [7]. Lymphangitis and lymphadenitis are not typically present [7]. In Wong et al.'s study, an intermediate stage of NF was characterized by the formation of small bullae [2]. The presence of such bullae filled with serous fluid was an important diagnostic clue. Late signs for their patients included large hemorrhagic bullae, skin necrosis, fluctuance, crepitus, and sensory and motor deficits [2]. Children usually present with fever, irritability, swelling of the affected area, possibly erythema, and pain out of proportion to clinical findings [17]. With infants it may be difficult to localize the pain while older children will tend to refuse to move the affected site [17].

The management of NF begins with early recognition. Unfortunately the diagnosis is often delayed due to a lack of definitive signs and symptoms. Some have tried to develop predictive tests for NF. Wong et al. propose a diagnostic scoring system, LINEREC (laboratory risk indicator for necrotizing fasciitis) that is based on numerous laboratory tests, including white cell count, electrolytes, and C-reactive protein which had a positive predictive value of 92.0% and negative predictive value of 96.0% [18]. More research needs to be done to evaluate whether such systems will be of benefit. Other modalities such as frozen-section biopsy, CT, MRI, and ultrasound can aid in diagnosis, but should never delay operative intervention [4, 19–21]. Management consists of early debridement, broad spectrum antibiotics against polymicrobial infection (usually penicillin or ampicillin, plus clindamycin, and an aminoglycoside), hemodynamic support, repeated examinations with debridement of the wound (usually within 24–36 h), and aggressive nutritional support [22–24]. Additionally, cultures should be obtained from blood and from the affected tissue. Another treatment which is still controversial is hyperbaric oxygen which may assist healing and prevent amputation [25, 26]. Despite intervention, necrotizing fasciitis has a mortality rate from 6 to 76%, with a review of 696 patients showing a rate of 34% [22, 27]. In the study by Wong et al., the only factor that was found to independently affect survival on multivariate logistic regression analysis was the delay in surgery [2]. This has been reinforced by numerous studies that show that early surgical debridement is associated with a significant decrease in mortality [23, 27, 28].

### 3.2. Group G Necrotizing Fasciitis

Group G Streptococci (GGS) are a part of the normal flora of human skin, pharynx, vagina, and gastrointestinal tract [29]. GGS are subdivided by colony size and hemolytic phenotype [10]. Isolates that produce large (≥5 mm) *β*-hemolytic colonies are morphologically similar to Group A Streptococcus and are classified as *S. dysgalactiae* subspecies *S. equisimilis* [10, 29]. Group G isolates that are small (≤5 mm) have variable hemolytic reactions and are classified within the *Streptococcus milleri *or *Streptococcus intermedius* group [10, 29]. GGS are not a common cause of bacteremia and have a relatively low mortality rate of about 8–16% [30, 31]. Other associated syndromes include pharyngitis, skin and soft tissue infections, pneumonia, septic arthritis, osteoarthritis, and endocarditis [29]. While invasive disease due to GGS is rare, it has been gaining increasing recognition in recent years. It usually occurs in patients with debilitating conditions [30]. One case, though, of neonatal sepsis caused by GGS in an infant without any known risk factors has been reported [32]. Necrotizing fasciitis due to a monomicrobial infection with GGS has been reported in 15 cases in the English literature (Table 1) [10–13, 16]. 

The characteristics of the patients with GGS NF are very similar to those with GAS NF. The patients with GGS NF were often elderly with an average age of 62 among the 14 previously reported cases. The only child was our 2-year-old patient. Ten patients had comorbid conditions, including diabetes and rheumatoid arthritis. Four cases had preceding trauma. The most common presenting signs and symptoms were swelling with redness, pain, and blister formation. The site of involvement was the leg in most cases, 1 case in the arm, and 1 case in the perineum. The organism was isolated from the site of involvement in all cases. All patients were treated with antibiotics, including cefazolin, cefazolin-clindamycin, piperacillin-tobramycin, penicillin-gentamicin, penicillin-flucloxacillin, piperacillin/tazobactam-tobramycin, carbapenem-minocycline,  penicillin-clindamycin,  and  flucloxacillin-gentamycin. Three patients received IVIG. Debridement was used in 12 cases, amputation in 1. Eight patients developed toxic shock syndrome. Toxic shock syndrome (TSS) is a rare occurrence with GGS, and its features and pathophysiology most likely are similar to that of GAS TSS [33]. Six of the patients with TSS died. Overall there were 6 deaths for a mortality of 40%. When compared with Group A Streptococci, the only significant difference was in the site of involvement. GAS may involve the extremities, trunk, or face while the patients with GGS were mainly affected in the lower extremities [11].

Our patient's comorbid condition was a vascular/lymphatic malformation. Venous malformations do present a known risk for streptococcal infection, even in otherwise healthy children [34]. There has even been a reported case of an arteriovenous malformation and necrotizing fasciitis [35]. In this case, though, it was a pulmonary arteriovenous malformation with resulting NF of the thigh. The mechanism proposed for this was the formation of septic emboli. In our patient, the malformation provided a direct nidus for infection. 

In summary, Group G Streptococcus is emerging as a cause of invasive infections like necrotizing fasciitis and toxic shock syndrome. The infections seem to follow a similar course to invasive infections caused by Group A Streptococci. Clinicians need to be aware of the possibility of such infections as early treatment is critical for survival, especially in children with extensive vascular malformations (a group who may have an increased risk of these infections similar to adult patients with peripheral vascular disease). 

## Figures and Tables

**Table 1:** Patients with GGS necrotizing fasciitis (taken from [10–13, 17], and current report).

Age/sex	Comorbidity	Site	Culture source	Therapy	Outcome
75/F	Syringomyelia	L leg	Tissue	Antibiotics, debridement	Survived
80/F	None	R leg	Tissue	Antibiotics, debridement	Survived (skin graft)
49/F	None	L ankle	Tissue	Antibiotics, debridement	Survived
75/M	None	R leg	Tissue	Antibiotics, debridement	Survived
71/M	None	L foot	Skin/bulla	Antibiotics, debridement	Survived (skin graft)
59/M	Not stated	R leg	Skin/blister	Antibiotics, debridement	Died
64/F	Diabetes mellitus	Both legs	Tissue	Antibiotics	Died
65/F	Rheumatoid arthritis	R arm	Blood/tissue	Antibiotics, amputation	Died
52/M	Diabetes mellitus	R leg	Tissue	Antibiotics, debridement	Survived
52/M	Diabetes mellitus	R leg	Tissue	Antibiotics, debridement	Survived
59/M	Hairy-cell leukemia	L leg	Blood/tissue	Antibiotics, debridement	Survived
58/M	Cirrhosis	L leg	Tissue	Antibiotics, debridement	Died
49/F	Rheumatoid arthritis	R leg	Blood/skin	Antibiotics	Died
48/M	None	R leg	Tissue	Antibiotics, debridement	Survived
77/F	Rheumatoid arthritis	R leg	Tissue/blood	Antibiotics	Died
2/M	Vascular/lymphatic malformation	Perineum	Tissue/stool	Antibiotics, debridement	Survived
